# Relationship between Massachusetts Youth Screening Instrument-second version and psychiatric disorders in youths in welfare and juvenile justice institutions in Switzerland

**DOI:** 10.1186/s12888-016-1032-1

**Published:** 2016-09-30

**Authors:** L. E. W. Leenarts, C. Dölitzsch, K. Schmeck, J. M. Fegert, T. Grisso, M. Schmid

**Affiliations:** 1 Kinder- und Jugendpsychiatrische Klinik Forschungsabteilung, Universitäre Psychiatrische Kliniken (UPK), Schanzenstrasse 13, 4056 Basel, Switzerland; 2 Klinik für Kinder- und Jugendpsychiatrie/Psychotherapie, Universitätsklinikum Ulm, Steinhövelstrasse 5, D-89075 Ulm, Germany; 3 Department of Psychiatry, University of Massachusetts Medical School, 55 Lake Avenue North, Worcester, MA 01655 USA

**Keywords:** Mental health screening, MAYSI-2, Psychiatric disorders, Juvenile justice, Gender

## Abstract

**Background:**

There is growing evidence that it is important to have well-standardized procedures for identifying the mental health needs of youths in welfare and juvenile justice institutions. One of the most widely used tools for mental health screening in the juvenile justice system is the Massachusetts Youth Screening Instrument-second version (MAYSI-2). To contribute to the body of research examining the utility of the MAYSI-2 as a mental health screening tool; the first objective of the current study was to examine the relationship between the MAYSI-2 and the Schedule for Affective Disorders and Schizophrenia for School-Age Children, Present and Lifetime version (K-SADS-PL) in a sample of Swiss youths in welfare and juvenile justice institutions using a cross-sectional design. Secondly, as the sample was drawn from the French-, German- and Italian-speaking parts of Switzerland, the three languages were represented in the total sample and consequently differences between the language regions were analyzed as well. The third objective was to examine gender differences in this relationship.

**Methods:**

Participants were 297 boys and 149 girls (mean age = 16.2, SD = 2.5) recruited from 64 youth welfare and juvenile justice institutions in Switzerland. The MAYSI-2 was used to screen for mental health or behavioral problems that could require further evaluation. Psychiatric classification was based on the Schedule for Affective Disorders and Schizophrenia for School-Age Children, Present and Lifetime version (K-SADS-PL). Binomial logistic regression analysis was used to predict (cluster of) psychiatric disorders from MAYSI-2 scales.

**Results:**

The regression analyses revealed that the MAYSI-2 scales generally related well to their corresponding homotypic (cluster of) psychiatric disorders. For example, the alcohol/drug use scale identified the presence of any substance use disorder and the suicide ideation scale identified youths reporting suicide ideation or suicide attempts. Several MAYSI-2 scales were also related to heterotypic (cluster of) psychiatric disorders. For example, the MAYSI-2 scale alcohol/drug use, was positively related to any disruptive disorder. Furthermore, the results revealed gender differences in the relationship between the MAYSI-2 and K-SADS-PL (e.g., in the boys’ subsample no MAYSI-2 scale was significantly related to any affective disorder; whereas, in the girls’ subsample the MAYSI-2 scales depressed-anxious and somatic complaints were significantly related to any affective disorder).

**Conclusions:**

Overall, The MAYSI-2 seems to serve well as a *first-stage screen* to identify service needs for youths in welfare and juvenile justice institutions in Switzerland. Its effectiveness to identify the presence of (cluster of) psychiatric disorders differs between genders.

## Background

Across multiple samples and settings, an extensive body of research has documented that a high proportion of youths involved in the juvenile justice system in Europe meet criteria for one or more psychiatric disorders (e.g., [1, 2]). For example, according to the epidemiological study of Dölitzsch et al. [3] 74 % of boys and girls in youth welfare and juvenile justice institutions (*n* = 483) in Switzerland meet criteria for psychiatric disorders, which include (among others); anxiety disorder, major depression, bipolar disorder, substance abuse and conduct disorder. These rates are even higher for girls than for boys. Scholars have documented that, compared to boys, girls were 1.4 times as likely to have at least one psychiatric disorder [4] and were significantly more likely to have comorbid disorders [5]. Moreover, the association between psychiatric disorders and the involvement in juvenile delinquency has been well established [5–7]. For example, youths who persistently abuse substances are significantly more likely to have conduct problems compared to non-users [8–10]. In addition, incarcerated girls report high levels of posttraumatic stress and depressive symptoms, which may be associated with their involvement in delinquent behavior [11]. Consequently, regardless of the nature of the association between psychiatric disorders and the involvement in juvenile delinquency; it is commonly recognized that these youths need treatment for their problems, and that the juvenile justice system should have adequate procedures for treating them [12]. However, a significant number of youths do not receive the appropriate treatment for their mental health problems [13, 14]. In this context, it is important to have well-standardized procedures for identifying the mental health needs of this vulnerable group as appropriate treatment-planning can only occur if reliable identification and description of youths’ mental health needs precede [15].

One of the most widely used tools for mental health screening in the juvenile justice system is the Massachusetts Youth Screening Instrument-second version (MAYSI-2; [16, 17]). The MAYSI-2 was specifically developed, normed and validated to identify youths entering the juvenile justice system in the United States of America (USA) with potential emotional or behavioral problems (e.g., suicidal and aggressive behavior) that could require further (psychiatric) evaluation [18]. The MAYSI-2 is accessible in its usage as it requires no more than 15 min to administer, uses low-cost materials, and requires no special clinical expertise to administer, score and interpret [16]. Subsequently, the MAYSI-2 is currently used in all detention, intake probation, and/or corrections facilities in about 44 states in the USA [18] and has shown to be reliable and valid in diverse samples of detained youths (e.g., [19–21]). For these reasons, there are currently systematic evaluations of implementing the MAYSI-2 into the juvenile justice system in Europe [22–24]. Although the MAYSI-2 was not developed to diagnose specific psychiatric disorders, its aim to screen for youths who may have psychiatric disorders indicates that MAYSI-2 scale scores are at least related to psychiatric disorders [21, 22, 25]. To our knowledge, the relationship between the MAYSI-2 scales and psychiatric disorders has only been studied in a few American and European samples of detained male and female youths (e.g., [21, 22, 26–28]). The results of these studies supported the construct validity of the MAYSI-2. It was found that some scales of the MAYSI-2 identified the presence of psychiatric disorders better than others; for example, the alcohol/drugs use scale of the MAYSI-2 was positively related to any substance use disorder. However, for girls, the traumatic experiences scale was not related to the any anxiety disorder cluster [21].

To further these studies, the primary purpose of the current study was to examine the relationship between the MAYSI-2 and the Schedule for Affective Disorders and Schizophrenia for School-Age Children Present and Lifetime version (K-SADS-PL) in a sample of Swiss youths in welfare and juvenile justice institutions using a cross-sectional design. Secondly, as the sample was drawn from the French-, German- and Italian-speaking parts of Switzerland, the three languages were represented in the total sample and consequently we could check for differences between the language regions. In view of the research suggesting that girls are more likely than boys to meet criteria for at least one psychiatric disorder or to have comorbid disorders [4, 5], the third objective of this study was to build on previous research by addressing possible gender differences in the relationship between the MAYSI-2 scales and the K-SADS-PL. Gaining greater insight into the utility of the MAYSI-2 is essential for optimizing the identification of mental health needs of youths admitted to the juvenile justice system in order to place them in appropriate treatment programs, and eventually help them to recover from their severe mental health problems [15].

## Methods

### Procedure

The current study was part of the larger *Swiss study for clarification and goal-attainment in youth welfare and juvenile justice institutions*, involving the standardized monitoring and evaluation of mental health problems of youths in welfare and juvenile justice institutions in Switzerland [29]. In our sampling procedure, *all* welfare and juvenile justice institutions with an official registration of the Swiss Federal Office of Justice (Bundesamt für Justiz, BJ) were invited to participate in the study. The BJ institutions represent institutions with different types of infrastructure (e.g., large versus small institutions, institutions with or without intern elementary schools, and internal versus external access to treatment programs). In addition, the BJ institutions reside a heterogeneous group of youths with even differences in youths between institutions (e.g., age, gender and reason for stay). However, the participating BJ institutions (35 %, *N* = 64) form a representative sample of the different types of institutions as well as of the youths who reside in them [29].

Adolescents who were admitted to one of the 64 facilities between 2007 and 2011 were asked to participate; with the exception of those who had a placement shorter than one month and those who, due to language problems, were not able to complete the French, German or Italian assessment tools. Adolescents and their primary caregivers were individually approached by trained staff of the institution who explained the aims and nature of the study. Following Swiss legislation, active informed consent was collected and, if the adolescent was younger than age 18, parental/primary caregiver informed consent was obtained as well.

The study was reviewed by the Ethics Review Committees of Basel, Lausanne (Switzerland) and Ulm (Germany). A total of 592 (32 %) adolescents from the 64 youth welfare and juvenile justice institutions, in the French- (20 facilities), German- (38 facilities) and Italian-speaking (6 facilities) parts of Switzerland were involved in the study.

The representativeness of the sample was checked by comparing the study sample with matched (i.e., on age and gender) adolescents who refused to participate in the study. The professional caregivers were asked to complete either the Child Behavior Checklist (CBCL; [30]) or the Young Adult Behavior Checklist (YABCL; [31]). CBCL or YABCL information was available for 94 % of all youths, the frequency of adolescents who scored in the clinical range on the *internalizing-*, *externalizing-* and *total problems scale* of the CBCL or the YABCL did not differ between both groups, which suggests that the sample was representative for youths in welfare and juvenile justice institutions in Switzerland. It is important to note that in Switzerland, youths can be placed in welfare and juvenile justice institutions because of: delinquent behavior (*criminal law measure*), youth welfare reasons (*civil law measure,* e.g., maltreatment, parental psychopathology, prostitution and drug abuse) or *other reasons* (e.g., their own or parents’ choice). These three groups currently reside in the same facilities.

### Participants

For the current study, data from 446 adolescents who completed both the MAYSI-2 and the Schedule for Affective Disorders and Schizophrenia for School-Age Children, Present and Lifetime version (K-SADS-PL) were analyzed. The adolescents’ ages ranged from 9 to 25 years (mean = 16.2, SD = 2.5). Among the 297 (66.6 %) boys and 149 (33.4 %) girls, 23.2 % were placed in the facility under a *criminal law measure*, 58.5 % under a *civil law measure* and 18.4 % because of *other reasons*. Of the total sample 14.8 % were from the French-speaking, 76.5 % from the German-speaking and 8.7 % from the Italian-speaking part of Switzerland. Most adolescents (83.9 %) were of Swiss nationality, with 77.1 % born in Switzerland and 22.9 % born in other countries.

Since differences between the French-speaking and Italian-speaking subsample were small (i.e., age, gender, reason for stay, country of birth, MAYSI-2 scores and percentage of adolescents with (cluster of) psychiatric disorders), and because of small sample sizes (details available upon request from the first author), the French/Italian-speaking subsamples were combined in the analyses.

### Assessment

#### Demographics

Background information (i.e., age, gender, reason for stay, language region, nationality and country of birth) was extracted by local staff from personal records. For the analyses the following variables were dichotomized: reason for stay (criminal law measure versus civil law measure/other reasons), language region (German versus French/Italian) and country of birth (Switzerland versus not Switzerland).

#### MAYSI-2

The French, German and Italian versions of the computerized MAYSI-2 [17] were used to screen for mental health or behavioral problems that could require further evaluation. It is important to note that when implementing the *Swiss study for clarification and goal-attainment in youth welfare and juvenile justice institutions* all youths residing in the institutions were asked to participate at one point of time, therefore all participating youths were screened with the MAYSI-2 at different time points after their facility intake (M = 18.5 months; SD = 21.9).

The MAYSI-2, designed specifically for use in the juvenile justice system, is a self-report questionnaire consisting of 52 items. The respondent rates the questions with *yes* (1 point) or *no* (0 points). The MAYSI-2 generates seven scales: alcohol/drug use, angry-irritable, depressed-anxious, somatic complaints, suicide ideation, thought disturbance and traumatic experiences. Of the seven scales, thought disturbance was not included in the current study, as it has been found to be a reliable scale only for boys. In addition, the thought disturbance scale would be related to *any schizophrenic or any other psychotic disorder*. However, only 5 youths were diagnosed as such so the n of this group was too small to include in the analyses. All of the scales, with the exception of the traumatic experiences scale, generate two types of cut-off scores: a ‘caution’ cut-off, to identify youths with a *clinically relevant* score; and a ‘warning’ cut-off, to identify youths *most in need of attention*. In the current study the ‘caution’ cut-off was used, as we were interested in identifying *all* youths with a possible basis for concern [16]. For the traumatic experiences scale, a cut-off score of 3 was used as it has been shown to be a promising cut-off to identify youths with symptoms of posttraumatic stress disorder (PTSD; [32]).

Earlier research on this questionnaire in juvenile justice samples displayed satisfactory psychometric properties [19, 33–35]. In the current study, Cronbach’s alpha coefficients of the scales ranged from .67 to .88, except for the traumatic experiences scale for boys (.59). An alpha lower than .60 can be considered insufficient [36]. In addition, adding the traumatic experiences scale for boys into the regression analyses did not reveal any significant results, nor did it change any of the other results. For these reasons the traumatic experiences scale for boys was not included in our final analyses (details available upon request from the first author).

#### K-SADS-PL

Psychiatric classification was based on the French [37], German [38] and Italian [39] translation of the K-SADS-PL [40]. The K-SADS-PL was administered by experienced psychologists. The psychologists were trained in a number of sessions in administering the K-SADS-PL by a child and adolescent psychiatrist and a psychologist who was experienced in the use of this instrument. During the study, the trained psychologists were monthly supervised in study team meetings. Agreement on ambiguous K-SADS-PL cases was reached in those team meetings. It is important to note that the trained psychologists were instructed to administer the K-SADS-PL interview as soon as possible (within 3 months) after the youths were screened with the MAYSI-2. However, due to conflicting schedules of youths, there could have been some exceptions.

The K-SADS-PL is a semi-structured interview used to assess a wide range of axis-I disorders. In the current study the K-SADS-PL was used to ascertain specific diagnoses (based on the 10^th^ revision of the International Statistical Classification of Diseases and Related Health Problems; ICD-10) grouped into five diagnostic clusters for the analyses: (1) *any substance use disorder* included abuse/dependence of alcohol and other substances; (2) *any affective disorder* included depressive, bipolar or manic disorders; (3), *any anxiety disorder* included obsessive-compulsive, generalized anxiety, phobic disorders, PTSD and acute stress reactions; (4) *any disruptive behavior disorder* included hyperkinetic, oppositional and conduct disorders and (5) *suicide ideation/suicide attempts* included thoughts about death, suicide ideation and suicidal actions.

The K-SADS-PL has been widely used in clinical settings and has displayed satisfactory psychometric properties in French [41–43], German [44] and Italian [45] child and adolescent psychiatric samples. In the current study, inter-rater reliability of the K-SADS-PL was assessed for the most prevalent disorder supplements (i.e., depression, attention deficit hyperactivity disorder, oppositional defiant disorder, conduct disorder, alcohol abuse and substance abuse). Cohen's kappa coefficients of these supplements were: .76 (depression), .87 (attention deficit hyperactivity disorder), .37 (oppositional defiant disorder), .43 (conduct disorder), 1.00 (alcohol abuse), and .86 (substance abuse).

### Statistics

First, we generated descriptive statistics (using Statistical Package for Social Science, SPSS, 21) for the study variables and compared demographics (i.e., age, gender, reason for stay, language region and country of birth), MAYSI-2 scores, and (cluster of) psychiatric disorders across language regions and gender via *t*-test and chi-square analyses.

Next, binomial logistic regression analysis was used to predict (cluster of) psychiatric disorders from MAYSI-2 scales. Although the MAYSI-2 was not developed to diagnose specific psychiatric disorders, its aim to screen for youths who may have psychiatric disorders indicates that MAYSI-2 scale scores are at least related to psychiatric disorders [21, 22, 25]. Following a study by Wasserman et al. [21], we similarly related MAYSI-2 scales to (cluster of) psychiatric disorders. As youths in welfare and juvenile justice institutions are likely to have comorbid disorders [5], MAYSI-2 scales were related to both homotypic and heterotypic (cluster of) psychiatric disorders. Relationships between MAYSI-2 scales and (cluster of) psychiatric disorders sharing the same underlying constructs were defined as homotypic mappings (i.e., relationships between disorders within a diagnostic grouping). Consequently; we related the MAYSI-2 scale alcohol/drug use to any substance use disorder, the MAYSI-2 scale angry-irritable to any disruptive behavior disorder, the MAYSI-2 scales depressed-anxious and somatic complaints to any affective and any anxiety disorder, and the MAYSI-2 scale suicide ideation to suicide ideation/suicide attempts. Note that the MAYSI-2 scale somatic complaints asks about bodily aches and pains associated with affective or anxiety disorders [16] and was therefore related to any affective and any anxiety disorder. Relationships between MAYSI-2 scales and (cluster of) psychiatric disorders not sharing the same underlying constructs were defined as heterotypic mappings (i.e., relationships between disorders from different diagnostic groupings). Consequently, we related the MAYSI-2 scales to all remaining, not-homotypic (cluster of) psychiatric disorders. Homotypic mappings are indicated with bordered cells in Table 2, Table 3 and Table 4.

As we were interested in differences between the language regions and possible gender differences in the relationship between the MAYSI-2 scales and the K-SADS-PL, we performed analyses on five separate subsamples: total sample, German-speaking subsample, French/Italian-speaking subsample, boys and girls.

In the regression analyses two groups of independent variables were entered simultaneously. The first group, which is only described in the tables, contained age, gender (not included in the gender subsample analyses), reason for stay, and language region (not included in the language region subsample analyses). In this way we adjusted for age, gender, reason for stay, and language region in the designated subsamples. The second group contained the MAYSI-2 scales.

## Results

### Descriptives and comparisons across subsamples (German-speaking and French/Italian-speaking, boys and girls)

The following percentages of youths scored at or above the ‘caution’ cut-off on one or more MAYSI-2 scale: alcohol/drug use (33 %), angry-irritable (51.8 %), depressed-anxious (50 %), somatic complaints (34.8 %), suicide ideation (41.9 %), traumatic experiences (girls only; 48.3 %) and 77.1 % scored at or above any ‘caution’ cut-off (excluding traumatic experiences). Of the total sample, 15 % were diagnosed with any substance use disorder, 15.9 % with any affective disorder, 10.8 % with any anxiety disorder, 47.1 % with any disruptive behavior disorder and 21.9 % with suicide ideation and suicide attempts.

Adolescents of the French/Italian-speaking subsample scored significantly higher than adolescents of the German-speaking subsample on the MAYSI-2 scales: angry-irritable, depressed-anxious and somatic complaints. Adolescents of the German-speaking subsample scored significantly higher than adolescents of the French/Italian-speaking subsample on the alcohol/drug use scale. No significant differences were found on the suicide ideation scale. Considering (cluster of) psychiatric disorders; a significantly higher proportion of adolescents of the French/Italian-speaking subsample had any affective disorder, any anxiety disorder, and suicide ideation and suicide attempts than adolescents of the German-speaking subsample. A significantly higher proportion of adolescents from the German-speaking subsample met criteria for any disruptive behavior disorder than adolescents from the French/Italian-speaking subsample. No significant differences were found between the language regions for any substance use disorder.

MAYSI-2 means were compared across gender and girls scored significantly higher than boys on the scales: angry-irritable, depressed-anxious, somatic complaints and suicide ideation. No significant differences were found on the alcohol/drug scale. Considering (cluster of) psychiatric disorders; girls were more likely than boys to meet criteria for any affective disorder, any anxiety disorder and suicide ideation and suicide attempts. Girls were less likely than boys to meet criteria for any disruptive behavior disorder. No significant differences were found for any substance use disorder (see Table 1 for comparisons across subsamples).

**Table 1:** Comparisons across subsamples (German-speaking and French/Italian-speaking, boys and girls)

Total sample (*n* = 446)						
	German-speaking subsample (*n* = 341)	French/Italian-speaking subsample (*n* = 105)	Comparison language regions	Boys (*n* = 297)	Girls (*n* = 149)	Comparison gender
MAYSI-2 scales	M (SD)	M (SD)		M (SD)	M (SD)	
Alcohol/drug use	2.74 (SD 2.81)	1.91 (2.30 SD)	t(208) = 3.04, *p* < .01	2.56 (SD 2.75)	2.51 (SD 2.67)	ns
Angry-irritable	4.25 (SD 2.74)	5.17 (2.42 SD)	t(193) = 3.29, *p* < .01	4.28 (SD 2.75)	4.84 (SD 2.55)	t(444) = 2.06, *p* < .05
Depressed-anxious	2.60 (SD 2.30)	4.16 (2.14 SD)	t(444) = 6.21, *p* < .001	2.44 (SD 2.12)	4.01 (SD 2.45)	t(262) = 6.70, *p* < .001
Somatic complaints	1.68 (SD 1.57)	2.88 (1.67 SD)	t(444) = 6.74, *p* < .001	1.54 (SD 1.45)	2.80 (SD 1.76)	t(252) = 7.53, *p* < .001
Suicide ideation	1.59 (SD 1.84)	1.52 (1.69 SD)	ns	1.25 (SD 1.66)	2.22 (SD 1.90)	t(264) = 5.32, *p* < .001
Traumatic experiences	–	–	–	–	–	–
Cluster of disorders (K-SADS-PL)	n (%)	n (%)		n (%)	n (%)	
Any substance use	53 (15.5 %)	14 (13.3 %)	ns	49 (16.5 %)	18 (12.1 %)	ns
Any affective	44 (12.9 %)	27 (25.7 %)	*χ*2(1) = 9.84, *p* < .01	39 (13.1 %)	32 (21.5 %)	*χ*2(1) = 5.16, *p* < .05
Any anxiety	19 (5.6 %)	29 (27.6 %)	*χ*2(1) = 40.63, *p* < .001	24 (8.1 %)	24 (16.1 %)	*χ*2(1) = 6.66, *p* < .05
Any disruptive behavior	189 (55.4 %)	21 (20.0 %)	*χ*2(1) = 40.44, *p* < .001	154 (51.9 %)	56 (37.6 %)	*χ*2(1) = 8.11, *p* < .01
Suicide ideation/suicide attempts	66 (19.4 %)	32 (30.8 %)	*χ*2(1) = 6.05, *p* < .05	49 (16.5 %)	49 (33.1 %)	*χ*2(1) = 15.87, *p* < .001

MAYSI-2 means and (cluster of) psychiatric disorders were compared separately across language regions and across gender

*MAYSI-2* Massachusetts Youth Screening Instrument-second version, *K-SADS-PL* Schedule for Affective Disorders and Schizophrenia for School-Age Children Present and Lifetime version, *ns* not significant

### Logistic regression

Results for the logistic regression analyses on the total sample (Table 2), showed that most homotypic mappings yielded significantly elevated odds ratios (ORs); with the exception of the somatic complaints scale. Youths in the total sample with a score at or above the caution cut-off on the MAYSI-2 scale somatic complaints were less likely to have any affective disorder. The depressed-anxious and somatic complaints scales revealed no significant OR for any anxiety disorder. Several heterotypic mappings also yielded significant ORs; the alcohol/drug scale revealed a significant OR for any anxiety disorder and for any disruptive disorder.

**Table 2:**
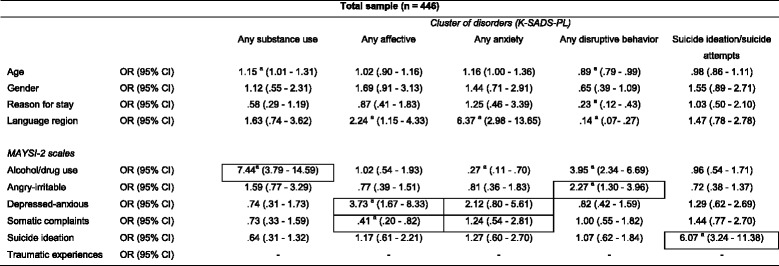
Logistic regression odds ratios for MAYSI-2 Scales (total sample)

As we did not include the traumatic experiences scale for boys (α = .59) in our analyses, we could not compute the logistic regression odds ratios for this scale for the total sample

Bordered cells represent homotypic mappings, others represent heterotypic mappings

*K-SADS-PL* Schedule for Affective Disorders and Schizophrenia for School-Age Children Present and Lifetime version, MAYSI-2 Massachusetts Youth Screening

Instrument-second version, *OR* odds ratio, *CI* confidence interval

^a^Significant OR at .05 level

The results of the logistic regression analyses on the German-speaking and the French/Italian-speaking subsample (Table 3) were comparable to the results of the total sample. Except for the German-speaking subsample, only the depressed-anxious scale revealed a significantly elevated OR for any affective disorder and no MAYSI-2 scale revealed a significantly elevated OR for any anxiety disorder. In the French/Italian-speaking subsample, the alcohol/drug use scale did not reveal a significantly elevated OR for any substance abuse disorder and the angry-irritable scale did not reveal a significant OR for any disruptive disorder when compared to the total sample.

**Table 3:**
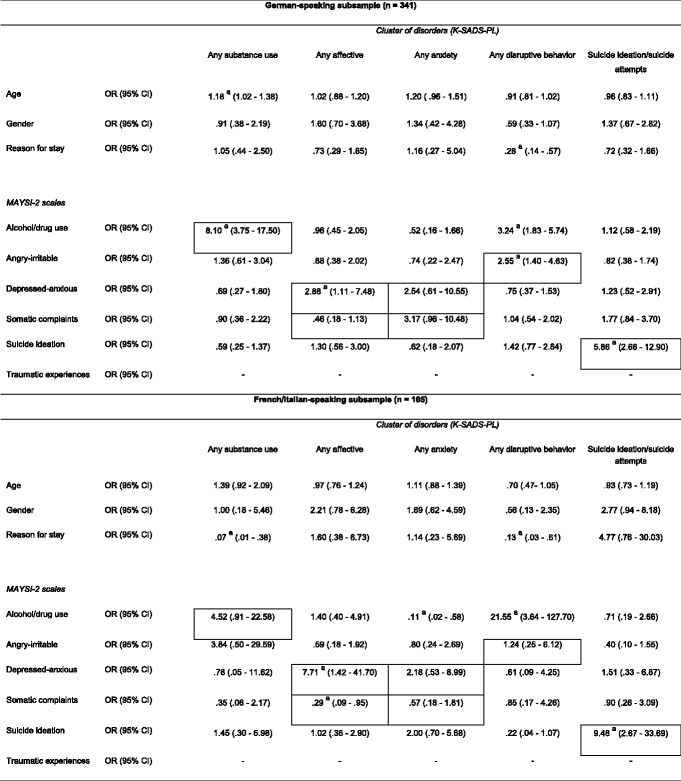
Logistic Regression Odds Ratios for MAYSI-2 Scales (German-speaking and French/Italian-speaking Subsample)

As we did not include the traumatic experiences scale for boys (α = .59) in our analyses, we could not compute the logistic regression odds ratios for this scale for the German-speaking and French/Italian-speaking subsample

Bordered cells represent homotypic mappings, others represent heterotypic mappings

*K-SADS-PL* Schedule for Affective Disorders and Schizophrenia for School-Age Children Present and Lifetime version, *MAYSI-2* Massachusetts Youth Screening Instrument-second version, *OR* odds ratio, *CI* confidence interval

^a^Significant OR at .05 level

The results predicting (cluster of) psychiatric disorders for boys (Table 4) showed that boys with a score at or above the caution cut-off on the MAYSI-2 scale alcohol/drug use were more likely to have any substance use disorder or to have any disruptive behavior disorder. However, boys with a score at or above the caution cut-off on the MAYSI-2 scale alcohol/drug use were less likely to have any anxiety disorder. Further, boys with a score at or above the caution cut-off on the MAYSI-2 scale suicidal ideation were more likely to have suicide ideation and suicidal actions. In the boys’ subsample, no MAYSI-2 scale revealed a significant OR for any affective disorder. The results predicting (cluster of) psychiatric disorders for girls (Table 4) showed that girls with a score at or above the caution cut-off on the MAYSI-2 scale alcohol/drug use or traumatic experiences were more likely to have any substance use disorder. Girls with a score at or above the caution cut-off on the MAYSI-2 scale depressed-anxious were more likely to have any affective disorder; whereas girls with a score at or above the caution cut-off on the MAYSI-2 scale somatic complaints were less likely to have any affective disorder. Further, girls with a score at or above the caution cut-off on the MAYSI-2 scales alcohol/drug use or angry-irritable were more likely to have any disruptive disorder. Girls with a score at or above the caution cut-off on the MAYSI-2 scale suicidal ideation were more likely to have suicide ideation and suicidal actions. In the girls’ subsample, no MAYSI-2 scale revealed a significant OR for any anxiety disorder.

**Table 4:**
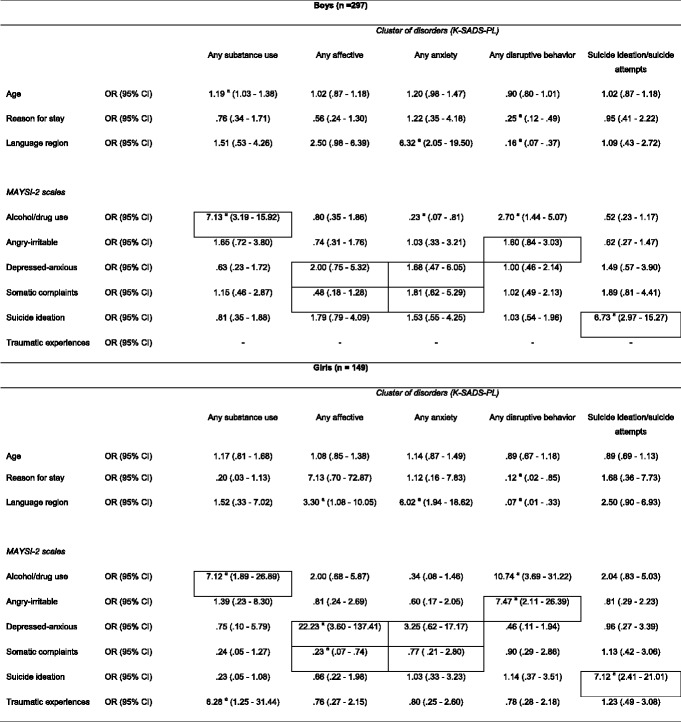
Logistic Regression Odds Ratios for MAYSI-2 Scales (Boys and Girls)

As we did not include the traumatic experiences scale for boys (α = .59) in our analyses, we could only compute the logistic regression odds ratios for this scale for the girls’ subsample

Bordered cells represent homotypic mappings, others represent heterotypic mappings

*K-SADS-PL* Schedule for Affective Disorders and Schizophrenia for School-Age Children Present and Lifetime version, *MAYSI-2* Massachusetts Youth Screening Instrument-second version, *OR* odds ratio, *CI* confidence interval

^a^Significant OR at .05 level

## Discussion

The primary purpose of the current study was to examine the relationship between the MAYSI-2 and the K-SADS-PL in a sample of Swiss youths (i.e., French, German and Italian language regions) in welfare and juvenile justice institutions using a cross-sectional design. The results of the analyses on the total sample are consistent with previous research [21, 22, 26–28] that has found the MAYSI-2 scales to be generally well-related to their corresponding homotypic (cluster of) psychiatric disorders. For example, the alcohol/drug use scale identified the presence of any substance use disorder, the angry-irritable scale related well to any disruptive behavior disorder and the suicide ideation scale identified youths reporting suicide ideation or suicide attempts. Surprisingly, within the total sample, those who scored at or above the caution cut-off on the MAYSI-2 scale somatic complaints were less likely to have any affective disorder. Comparable results were found in the French/Italian-speaking and girls’ subsamples. In the German-speaking and boys’ subsamples the somatic complaints scale revealed no significant OR for any affective disorder. This finding is difficult to interpret, but it may be explained by the possibility that Swiss youths mention their actual physical illnesses on the somatic complaints scale rather than bodily aches and pains associated with affective or anxiety disorders. Additional research is needed to investigate the predictive value of the somatic complaints scale in other (language) subsamples.

Further, of the total sample, youths who scored at or above the caution cut-off on the MAYSI-2 scale alcohol/drug use were less likely to have any anxiety disorder. Comparable results were found in the French/Italian-speaking and boys’ subsamples. An explanation may be that, in some youths, alcohol/drug use helps to reduce symptoms of anxiety, and subsequently an anxiety disorder is difficult to detect. Additionally, in the total sample, the MAYSI-2 scales depressed-anxious and somatic complaints did not predict any anxiety disorder. Comparable results were found in all the other subsamples (i.e., German-speaking and French/Italian-speaking subsample, boys and girls). An explanation for this finding may be that the variable language region influenced the relationship between the depressed-anxious and somatic complaints scales, and any anxiety disorder. This reasoning is supported by the fact that when the variable language region (which contributed strongly to any anxiety disorder) was left out of the regression analysis, the depressed-anxious scale contributed significantly to any anxiety disorder. In further support of this reasoning, we found that the correlations between the depressed-anxious and somatic complaints scales, and any anxiety disorder were higher than the (negative) correlation between the alcohol/drug scale and any anxiety disorder. However, the correlations between the depressed-anxious and somatic complaints scales, and language region were higher than the correlations between both scales and any anxiety disorder. As mentioned above it may be that Swiss youths mention their actual physical illnesses on the somatic complaints scale rather than bodily aches and pains associated with affective or anxiety disorders. In addition, in the Wasserman study [21] an elevated OR was found for the depressed-anxious scale for any affective and any anxiety disorder (the somatic complaints scale was not included in the analyses). Consequently, another explanation may be that, the aim of the depressed-anxious scale (i.e., reveal depressed or anxious moods and problems) is less well covered in the translated versions of the MAYSI-2.

Furthermore, and consistent with Wasserman et al. [21], several MAYSI-2 scales were related to heterotypic (cluster of) psychiatric disorders. For example, in the total sample the MAYSI-2 scale alcohol/drug use, was positively related to any disruptive disorder. Comparable results were found in all the other subsamples (i.e., German-speaking and French/Italian-speaking subsample, boys and girls). An explanation for this may be found in the co-occurrence of substance abuse with disruptive disorders, as it is well documented [8–10] that disruptive disorders in youths are significantly related to problems associated with drug and alcohol abuse.

Overall, the MAYSI-2 scales related well to their corresponding homotypic (cluster of) psychiatric disorders in the German-speaking and French/Italian-speaking subsamples. Although small discrepancies between both samples (e.g., relationship between MAYSI-2 scales, and any affective and any anxiety disorder) were observed, the MAYSI-2 seems to serve well as a *first-stage screen* to identify service needs for youths in welfare and juvenile justice institutions in the French-, German- and Italian-speaking parts of Switzerland.

The third purpose of the current study was to build on previous research by addressing gender differences in the relationship between the MAYSI-2 scales and (cluster of) psychiatric disorders. The results of the analyses on the boys’ subsample showed that no MAYSI-2 scale was significantly related to any affective disorder. In addition, the MAYSI-2 scale angry-irritable was not significantly related to any disruptive behavior disorder. As the identification of (cluster of) psychiatric disorders in juvenile justice youths is influenced by gender variations in symptom expression (boys tend to reveal their feelings on self-report scales less readily than girls; [16]), it may be reasonable to suggest that the current caution cut-off scores for boys under-detect certain disorders. Furthermore, the traumatic experiences scale of the MAYSI-2 was developed to indicate only the degree to which youths had been exposed to traumatic experiences and not to indicate the presence of a psychiatric disorder [16]. It is noteworthy that in the girls’ subsample, the traumatic experiences scale significantly predicted any substance abuse disorder. Prior research has documented that traumatization and victimization experiences may be a risk factor in girls in understanding disruptive and substance abusing behavior [46, 47]. Therefore, this finding may indicate that the MAYSI-2 scale traumatic experiences is able to identify substance abusing behavior associated with exposure to traumatic experiences in this subsample. The MAYSI-2 scale suicide ideation was significantly related to suicide ideation/suicide attempts in both the boys’ and girls’ subsample. This finding is in line with one of the aims of the MAYSI-2, which is to identify youths who may be a danger to themselves and are in need of *direct* attention [16].

Several limitations should be mentioned. First, we should note that several findings of the current study should be interpreted with caution as low power (due to the relatively small number of girls (*n* = 18) and youths from the French/Italian-speaking subsample (*n* = 14) diagnosed with any substance use disorder) may have influenced the results. In addition, the inter-rater reliability of the oppositional defiant disorder and conduct disorder supplement of the K-SADS-PL was relatively low and may have influenced the results as well. Several explanations for the low inter-rater reliability could be the case; one explanation may be that, juveniles with a very clear/obvious ODD or CD diagnosis may have refused to get their interviews taped. Also, about 60 % of our sample fulfilled criteria for more than one diagnosis, the high co-morbidity may have reduced the inter-rater reliability for specific diagnoses. Another explanation may be that the different languages (i.e., French, German and Italian) of the interviewers influenced the inter-rater reliability. However, agreement on ambiguous K-SADS-PL (real) cases was reached in study team meetings.

Another limitation is that, when scoring the MAYSI-2 norm scores of juvenile justice youths in the USA [16] were used and juvenile justice populations, due to differences in juvenile justice systems and policies, may not be similar in Switzerland and the USA. Furthermore, the MAYSI-2 is a mental health screening tool created for youths between 12 and 17 years of age, and approximately 20 % of the present sample was 18 years of age or older. Although previous research [22] has shown that the psychometric properties of the MAYSI-2 are well supported in this age group, future studies are needed to test whether the relationship between the MAYSI-2 and (cluster of) psychiatric disorders are similar across different age groups. Lastly, due to the study design and due to conflicting schedules of youths; the time that passed between facility intake and the MAYSI-2 screening, and the time that passed between the MAYSI-2 screening and the K-SADS-PL interview was different for all youths and could have influenced the results.

Despite these limitations, the current study leads us to formulate a number of recommendations for future research. First, since the prevalence of traumatization and victimization among boys in youth welfare and juvenile justice institutions should not be overlooked [48], future research should investigate whether the traumatic experiences scale of the MAYSI-2 is able to identify the presence of (cluster of) psychiatric disorders associated with exposure to traumatic experiences in boys. In addition, with regard to the discussion about a new diagnosis to capture what chronically trauma exposed youths suffer from (i.e., *complex PTSD* or *developmental trauma disorder*) [47, 49] it would be usefull to investigate whether the traumatic experiences scale of the MAYSI-2 is able to identify this potential new diagnosis. Second, as in some cases the variable language region seemed to influence the relationship between the MAYSI-2 and the K-SADS-PL (i.e., between the depressed-anxious and somatic complaints scales, and any anxiety disorder); the results of this study indicate the need to test the relationship between the MAYSI-2 and the K-SADS-PL more elaborately in larger differentiated language samples in Europe. Lastly, as comorbidity is common in youths in welfare and juvenile justice institutions [3, 4, 50], and difficult to detect, future research should explicitly test whether the MAYSI-2 is able to distinguish comorbid (cluster of) psychiatric disorders in these youths.

## Conclusions

Our study shows that several MAYSI-2 scales were significantly related to homotypic and heterotypic (cluster of) psychiatric disorders in boys and girls in youth welfare and juvenile justice institutions in Switzerland. These relations were found to differ across language regions and across gender. In addition, our study shows that the MAYSI-2 is able to identify youths who may be a danger to themselves and are in need of *direct* attention (i.e., youths with suicidal ideation). However, some expected relations were not present (e.g., MAYSI-2 scale depressed-anxious and any anxiety disorder), or harder to interpret (e.g., MAYSI-2 scale somatic complaints and any affective disorder). Overall, the current study contributes to the body of research examining the utility of the MAYSI-2 as a mental health screening tool for youths in welfare and juvenile justice institutions. Psychiatric disorders in youths in welfare and juvenile justice institutions are considerable [1–3] and appear to be related to one and another (e.g., [8–10, 46, 47]). Although the separate MAYSI-2 scales were developed to screen for the presence of their corresponding homotypic (cluster of) psychiatric disorders; clinicians and practitioners should also, in their clinical practice, pay attention to the heterotypic (cluster of) psychiatric disorders which may be associated with an elevated score on a certain MAYSI-2 scale.

## Abbreviations

CBCL: Child Behavior Checklist
CI: Confidence interval
ICD-10: International Statistical Classification of Diseases and Related Health Problems, 10^th^ revision
K-SADS-PL: Schedule for Affective Disorders and Schizophrenia for School-Age Children, Present and Lifetime version
MAYSI-2: Massachusetts Youth Screening Instrument - second version
OR: Odds ratio
PTSD: Posttraumatic stress disorder
SPSS: Statistical Package for Social Sciences
USA: United States of America
YABCL: Young Adult Behavior Checklist
